# Unilateral Absence of the Pulmonary Artery

**DOI:** 10.5334/jbsr.1611

**Published:** 2018-11-28

**Authors:** Aliaksandr Anisau, Filip Vanhoenacker, Ivan Pilate

**Affiliations:** 1 AZ Sint-Maarten Mechelen/Duffel, BE; 2 AZ Sint-Maarten and University (Hospital) Antwerp/Ghent, BE

**Keywords:** absence pulmonary artery, tracheal bronchus, CT

A 37-year-old man underwent computed tomography (CT) of the neck because of laryngeal pain he had experienced for 10 weeks, with normal clinical and endoscopic findings. CT revealed a displacement of the trachea and the larynx to the right. The images of the partially scanned upper thorax showed anomalies in the right lung: a tracheal bronchus to the apical segment of the upper lobe, interstitial abnormalities and volume loss of the right lung.

Medical history revealed that the patient always had lower than average stamina and experienced respiratory problems soon after birth, without any precise diagnosis.

A subsequent contrast-enhanced CT of the chest was performed, depicting thickened intralobular and interlobular septa (Figure 1), a tracheal bronchus (Figure 2) and absence of the right pulmonary artery (Figure 3). The arterial blood supply to the right lung was provided by hypertrophic bronchial arteries (Figure 3) and branches of the coeliac trunk and abdominal aorta (not shown). The left lung was hyperinflated.

**Figure 1:**
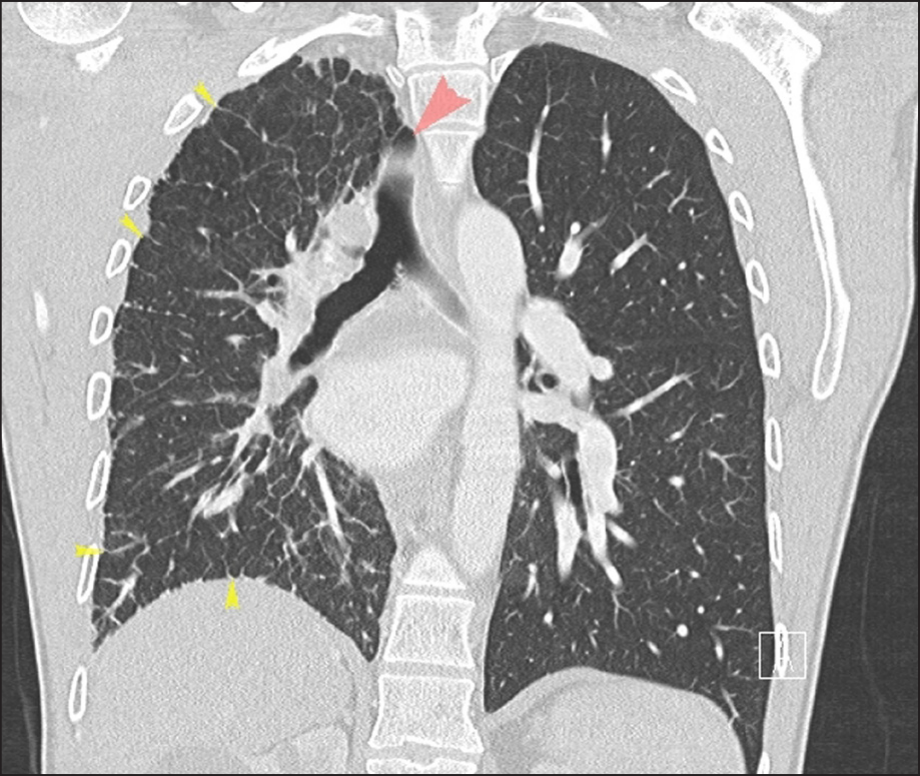
CECT of the thorax, lung window, coronal reformatted image. Hyperinflation of the left lung, smaller volume of the right lung. Mediastinal shift to the right side (red arrow pointing to displaced trachea). Thickened intralobular and interlobular septa (yellow arrows).

**Figure 2:**
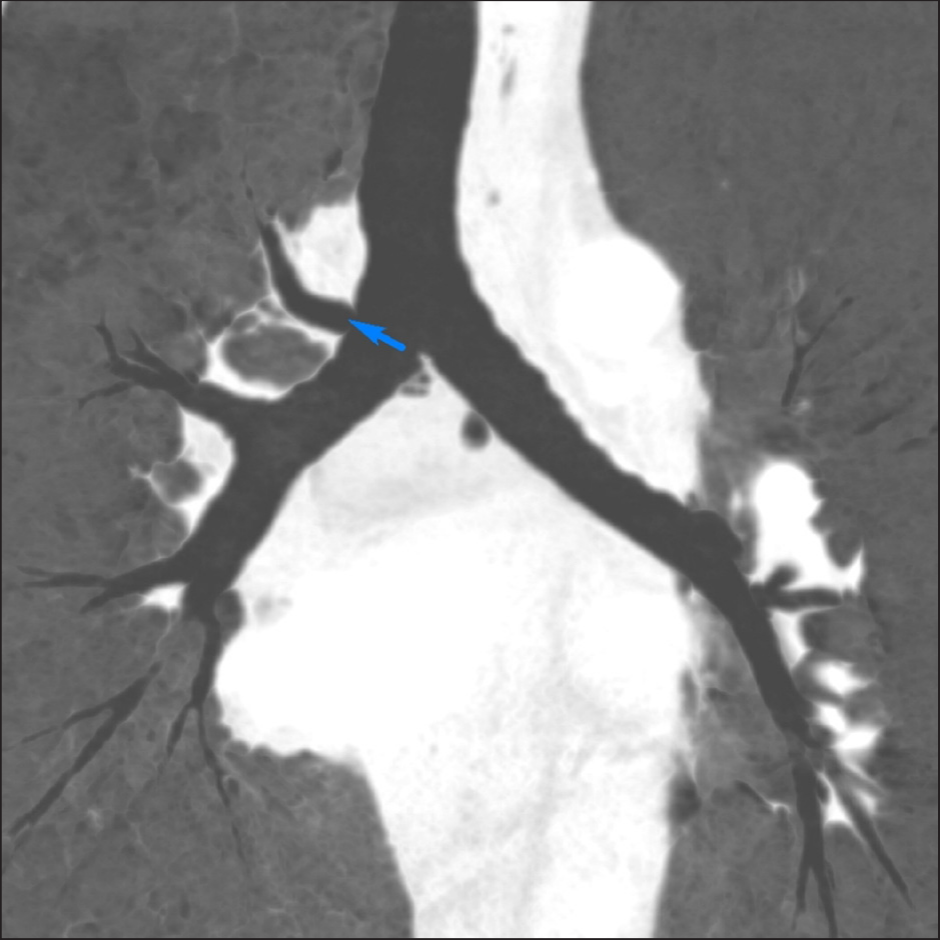
CECT of the thorax, lung window, coronal MinIP reconstruction. Tracheal bronchus arising at the right side of the carina (blue arrow).

**Figure 3:**
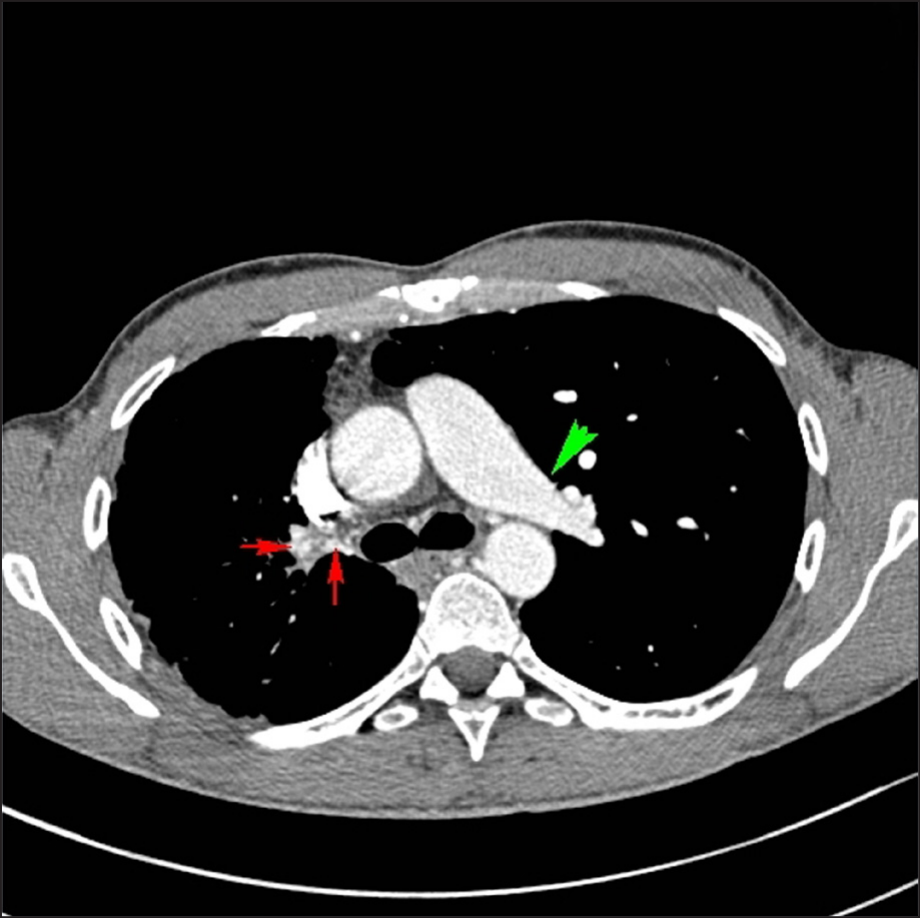
CECT of the thorax, soft tissue window, axial image. Normal appearance of the left pulmonary artery (green arrow). Absent right pulmonary artery. Prominent bronchial arteries supplying the right lung (red arrows). Smaller volume of the right lung.

The patient underwent an ultrasonography of the heart, which revealed no congenital cardiac anomalies and no signs of pulmonary hypertension.

## Comment

Unilateral absence of the pulmonary artery(UAPA) that normally arises from the sixth aortic arch is a very rare congenital malformation with a prevalence of 0.0005%. It may be isolated, but it is more often associated with other congenital cardiac defects such as aortic coarctation, tetralogy of Fallot, atrial septal defect, patent ductus arteriosus, truncus arteriosus, right aortic arch and pulmonary atresia [1]. UAPA is more common on the right side [1]. UAPA may present in infancy with respiratory distress, pulmonary hypertension and congestive heart failure [1]. When severe pulmonary hypertension does not develop in infancy, the condition may remain asymptomatic until adulthood. Adults with isolated UAPA can present with recurrent respiratory infections, exercise intolerance and haemoptysis [1]. Haemoptysis is caused by excessive collateral circulation, may be self-limiting or massive and life-threatening. A chest radiography, often performed because of recurrent respiratory infections, typically shows an ipsilateral small hemithorax, diminished hilar vasculature, mediastinal shift to the affected side, and elevation of hemidiaphragm [1]. When these findings are present, a contrast-enhanced CT scan of the chest should be performed, which will depict clearly the absent pulmonary artery, the collateral circulation and possibly an associated congenital defect. A cardiac ultrasonography should always be performed to rule out congenital heart defects and pulmonary hypertension.

The collateral circulation can arise from bronchial, intercostal, subdiaphragmatic, subclavian or even coronary arteries. The thickened intralobular and interlobular septa result from collateral circulation, which should not be misinterpreted as interstitial fibrosis. These interstitial changes arise due to chronic high pressures, which are caused by the left-to-right shunt.

There is no known embryological link between UAPA and a tracheal bronchus, the latter was most likely an incidental finding in this case. Both conditions predispose to recurrent respiratory infections.
